# Diagnostic accuracy of noninvasive fractional flow reserve derived from computed tomography angiography in ischemia-specific coronary artery stenosis and indeterminate lesions: results from a multicenter study in China

**DOI:** 10.3389/fcvm.2023.1236405

**Published:** 2023-10-02

**Authors:** Yaodong Ding, Quan Li, Yang Zhang, Yida Tang, Haitao Zhang, Qing Yang, Xiling Shou, Yicong Ye, Xiliang Zhao, Yi Ye, Chao Zhang, Yuqi Liu, Yong Zeng

**Affiliations:** ^1^ Department of Psychology, The University of Hong Kong, Hong Kong, Hong Kong SAR, China; ^2^ Department of Cardiology, Peking University Third Hospital, Beijing, China; ^3^ Department of Cardiology, Chinese Academy of Medical Sciences, Fuwai Hospital, Beijing, China; ^4^ Department of Cardiology, Tianjin Medical University General Hospital, Tianjin, China; ^5^ Department of Cardiology, Shanxi Provincial People’s Hospital, Shanxi, China; ^6^ Shenzhen Escope Technology Ltd., Shenzhen, China

**Keywords:** coronary computed tomography-derived fractional flow reserve (CT-FFR), computational fluid dynamics (CFD), computed tomography (CTA), coronary artery disease (CAD), indeterminate lesions

## Abstract

**Background:**

To determine the diagnostic performance of a novel computational fluid dynamics (CFD)-based algorithm for *in situ* CT-FFR in patients with ischemia-induced coronary artery stenosis. Additionally, we investigated whether the diagnostic accuracy of CT-FFR differs significantly across the spectrum of disease and analyzed the influencing factors that contribute to misdiagnosis.

**Methods:**

Coronary computed tomography angiography (CCTA), invasive coronary angiography (ICA), and FFR were performed on 324 vessels from 301 patients from six clinical medical centers. Local investigators used CCTA and ICA to conduct assessments of stenosis, and CT-FFR calculations were performed in the core laboratory. For CCTA and ICA, CT-FFR ≤ 0.8 and a stenosis diameter ≥ 50% were identified as lesion-specific ischemia. Univariate logistic regression models were used to assess the effect of features on discordant lesions (false negative and false positive) in different CT-FFR categories. The diagnostic performance of CT-FFR was analyzed using an invasive FFR ≤ 0.8 as the gold standard.

**Results:**

The Youden index indicated an optimal threshold of 0.80 for CT-FFR to identify functionally ischemic lesions. On a per-patient basis, the diagnostic sensitivity, specificity, accuracy, positive predictive value (PPV), and negative predictive value (NPV) for CT-FFR were 96% (91%–98%), 92% (87%–96%), 94% (90%–96%), 91% (85%–95%), and 96% (92%–99%), respectively. The diagnostic efficacy of CT-FFR was higher than that of CCTA without the influence of calcification. Closer to the cut point, there was less certainty, with the agreement between the invasive FFR and the CT-FFR being at its lowest in the CT-FFR range of 0.7–0.8. In all lesions, luminal stenosis ≥ 50% significantly affected the risk of reduced false negatives (FN) and false positives (FP) results by CT-FFR, irrespective of the association with calcified plaque.

**Conclusions:**

In summary, CT-FFR based on the new parameter-optimized CFD model has a better diagnostic performance than CTA for lesion-specific ischemia. The presence of calcified plaque has no significant effect on the diagnostic performance of CT-FFR and is independent of the degree of calcification. Given the range of applicability of our software, its use at a CT-FFR of 0.7–0.8 requires caution and must be considered in the context of multiple factors.

## 1. Introduction

As a noninvasive assessment tool, computed tomography (CTA) is widely used in the screening of coronary artery disease (CAD) (1, 2). However, as CTA only provides an imaging assessment of the degree of stenosis, it has many limitations in assessing the physiological function of lesions. Indeed, CTA often underestimates or overestimates the functional severity of lesions, particularly in intermediate and multivessel lesions, which cause reduced coronary blood flow and myocardial ischemia (3, 4). The invasive flow reserve fraction (FFR) is the current clinical standard for determining the hemodynamic significance of CAD (5). The FFR is regarded as a powerful tool for identifying patients with CAD who are likely to benefit from revascularization and to reduce the rate of composite end points (6, 7).

Coronary computed tomography-derived fractional flow reserve (CT-FFR) using computational fluid dynamics (CFD) has been proposed and validated in prospective clinical trials as a non-invasive technique to identify ischemic and non-ischemic lesions (8). The DeFACTO, DISCOVER-FLOW, and HeartFlow NXT trials have shown that CT-FFR improves diagnostic accuracy compared to CTA alone (9–11). Although it is important to understand the overall diagnostic accuracy of CT-FFR, physicians must also know the specific value of CT-FFR, as well as its positive and negative predictive value. In the PROMISE study, a CT-FFR < 0.80 was more predictive of revascularization or major adverse cardiovascular events than severe stenosis on CCTA in patients with stable chest pain, while CT-FFR values of >0.90 and ≤0.60 provided almost complete certainty (12, 13). However, further analysis of these lesions has been limited.

Recently, a novel CFD-based CT-FFR algorithm has been developed to provide accurate and rapid FFR values based on a three-dimensional, full-field hydrodynamic simulation of the entire coronary tree. This fully automated solution eliminates the need for external imaging specialists, reduces processing time, and improves diagnostic accuracy as it eliminates the potential for human error and reduces inter-observer variability. The objective of this multicenter clinical trial in China was to evaluate the diagnostic performance of CT-FFR for detecting ischemia with FFR as a reference, to determine whether the diagnostic accuracy of CT-FFR varies significantly across the spectrum of conditions, and to establish the level of confidence that the physician has that the findings are on a given side of a clinical decision threshold.

## 2. Methods

### 2.2. Study population

This multicenter study enrolled 397 patients with suspected CAD undergoing CCTA with at least one luminal diameter stenosis (DS) of 30%–90% documented between May 2020 and June 2020 at six Chinese medical centers. The studies involving human participants were reviewed and approved by the Ethics Committees of Anzhen Hospital. Subjects who met the exclusion criteria underwent CCTA at the study center within 30 days before invasive coronary angiography (ICA). Subjects who had undergone CCTA, and whose image quality met the requirements, at the study center underwent ICA and FFR with pressure guidewire measurement. Criteria for Inclusion of CCTA Imaging: (I) CCTA should be performed on a device with at least 64 detector rows; (II) The CCTA image is clearly readable; (III) 30% to 90% stenosis of the coronary lesion diameter as shown by CCTA imaging; (IV) The reference vessel diameter of the stenotic coronary lesion is ≥2 mm as shown by CCTA imaging.The general exclusion criteria were as follows: (I) patient age <18 or >80; (II) pregnant or lactating women; (III) allergy to contrast agent or adenosine; (IV) previous myocardial infarction within 30 days before CCTA examination; (V) previous coronary artery bypass grafting (CABG), stent, pacemaker placement, implantable cardioverter defibrillator (ICD), or prosthetic valve; (VI) hypertrophic obstructive cardiomyopathy or severe heart failure (NYHA ≥ III); (VII) body mass index >35 kg/m^2^; (VIII) non-signed informed consent and (IX) Abnormalities in liver and kidney function (values exceeding three times the reference value). The study was approved by the ethics committee of each of the participating medical centers, and all patients provided written informed consent.

### 2.2. CTA acquisition

**Figure 1:**
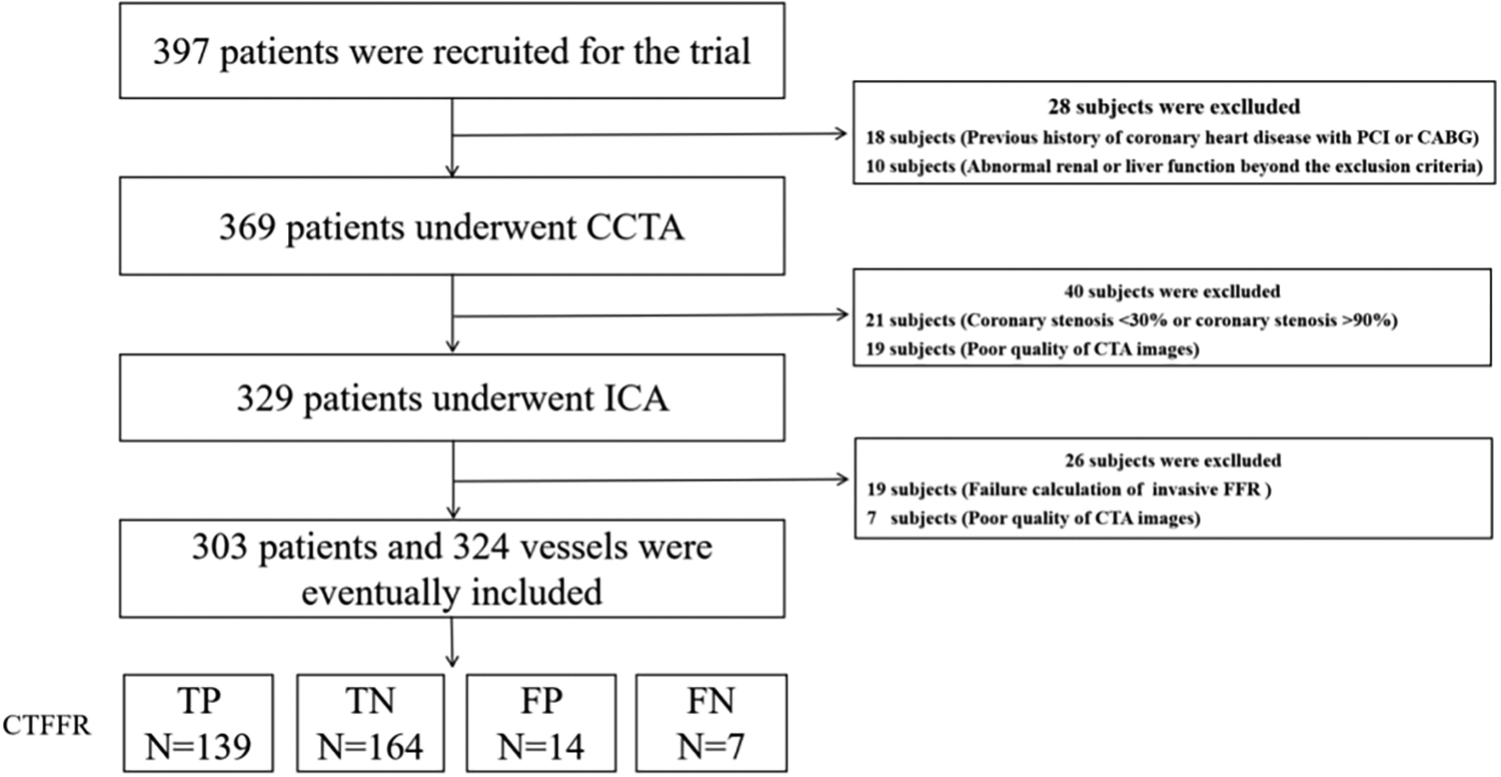
Study enrolment. CCTA, coronary computed tomography angiography; CT-FFR, coronary computed tomography angiography derived fractional flow reserve; FFR, fractional flow reserve.

For the CT image acquisition, multidetector scanners from three leading manufactures (Somatom Definition, Siemens, Forchheim, Germany; Aquilion One, Toshiba, Otawara, Japan; Optima CT660, GE Healthcare, Milwaukee, WI), each with detectors more than 64-rows, were used. CTA was performed in accordance with the standard protocol, and prospective triggering was used for scan acquisition (Figure 1A). The core laboratory followed the quality standards as defined in the guidelines. All CCTAs were acquired using electrocardiogram (ECG)-triggered adaptive sequences. The scan parameters were as follows: tube voltage, 100–120 kV; tube current, 100–300 mAs; collimator width, 128 × 0.625 mm; x-ray tube speed, 0.27/s/rev; and matrix, 512 × 512. Next, 50–70 ml of nonionic contrast agent (Iophorol, 370 mg/ml, Jiangsu Hengrui) was injected at a flow rate of 4–5 ml/s, followed by 50-ml saline at the same flow rate. The ascending aorta at the level of the lung window was set as the dynamic monitoring region, and the scanning was automatically started with a delay of 6 s after the CT value reached 150 HU. Any stenosis of ≥50% was considered significant by angiography. In 303 patients, coronary artery calcification was quantified using Agatston scores (AS), which were summed for all coronary arteries to obtain the total coronary calcification.

### 2.3. CT-FFR based on computational fluid dynamics

**Figure 2:**
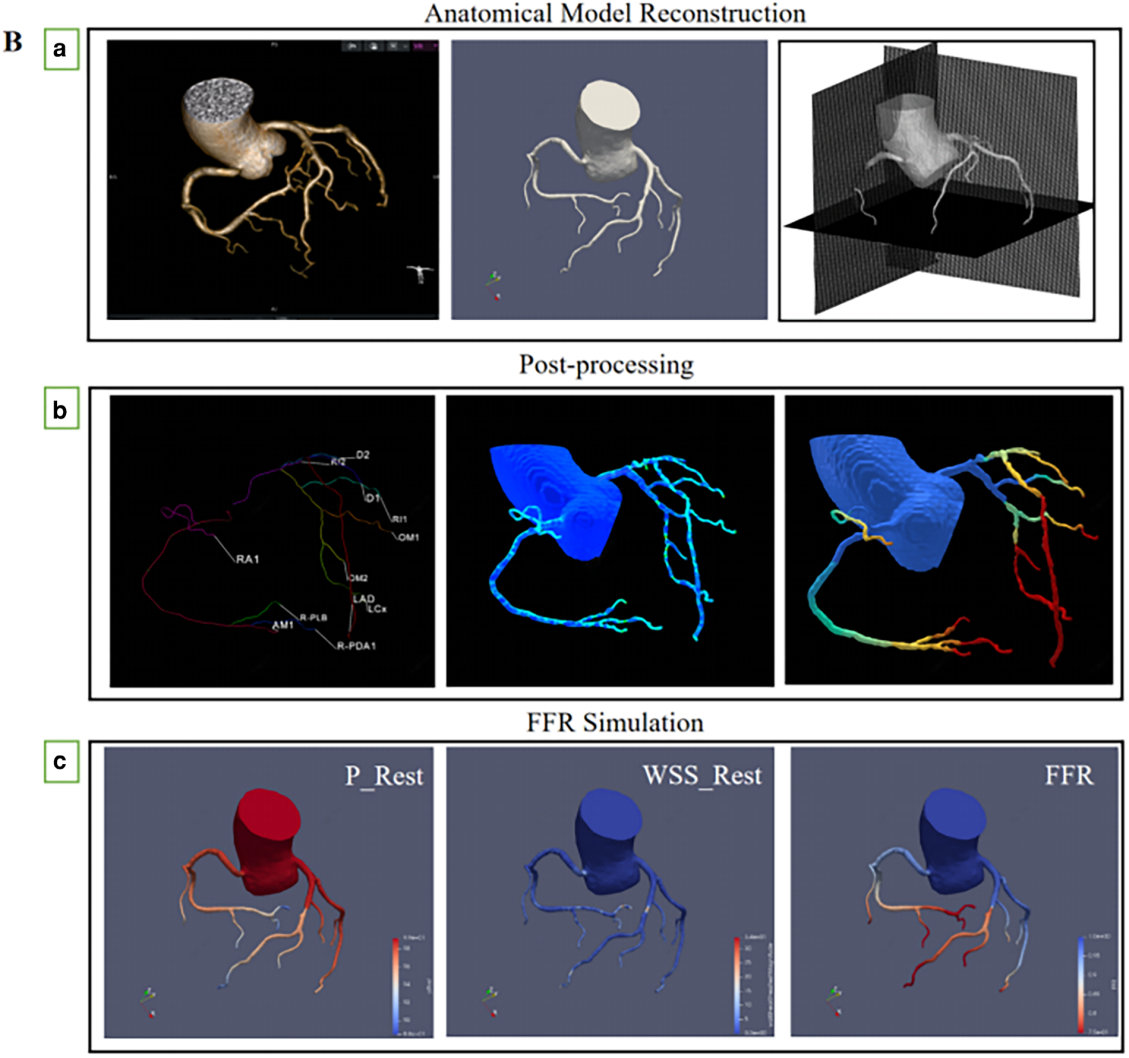
(**A**) multiplanar reformatting of (**a**) coronary CT angiography, (**b**) CT-FFR of the right coronary artery system, and CT-FFR value (0.886) was measured (**c**) invasive coronary angiography, (**d**) invasive FFR measurements, the FFR measured at the corresponding position was 0.88. (**B**) (**a**) Routine coronary computed tomography angiography are received and reconstruction of anatomical model, (**b**) Coronary artery physiological models were measured at corresponding locations, (**c**) The physical laws of fluid dynamics are used to calculate coronary blood flow, and calculation of fractional flow reserve from a standard acquired coronary computed tomography datasets.

CT-FFR measurement was computed using CAscope (EScope Ltd., Shenzhen, China). CAscope adopted a deep learning method for vessel centerline extraction, which ensured fast and complete coronary vessel tree construction with minimal user intervention. CT-FFR calculation was performed by core laboratory investigators in a blinded manner, according to the following steps (Figure 2B).

#### 2.3.1. 3D coronary anatomical models simulating maximal hyperemia

The three-dimensional (3D) surface model of the vessel lumen was created using a skeleton shape model-guided level set method; this new method uses a local adaptive threshold inferred from an artificial intelligence (AI) model to guide the level set method. This new deep neural network minimizes the chance of small vessel branches breaking into disconnected fragments by introducing a region-growth-like loss function that severely penalizes any topological changes in the predicted outcome compared to the underlying factual segmentation labels (Figure 2Ba).

#### 2.3.2. Definition of luminal centerline and boundary

The blood flow was simulated by solving Navier-Stokes equations using an in-house GPU-accelerated code based on the framework of the immersed boundary method. To minimize host-device communication, memory for the arrays that store pressure and velocities were allocated to the global device memory. Boundary conditions were transferred to GPU devices once throughout the simulation (Figure 2Bb).

#### 2.3.3. CT-FFR calculation

Blood flow was simulated by solving the Navier-Stokes equations using the CFD module of the CAscope software. The GPU-accelerated algorithm is based on the Immersed Boundary Method framework (14). First, to predict the velocity field and to update the right-hand side of the pressure Poisson equation, the modified momentum equations were solved using the iterative method. Then, the BiCGStab solver was used to solve the pressure Poisson equation. A parallel reduction algorithm was used to optimize the BiCGStab point products. Finally, the velocity field was corrected based on the resultant pressure values. These steps were repeated until convergence was achieved to obtain the hemodynamic results of the vessels. Flow-limiting lesions were identified as CT-FFR ≤ 0.80 (Figure 2Bc).

### 2.4. ICA and FFR

ICA and FFR measurements were performed according to the standard guidelines. FFR was assessed in at least one vessel with a diameter ≥2.0 mm and stenosis ≥30% during ICA. Intracoronary (40–60 μg/kg min) or intravenous (140–180 μg/kg min) adenosine infusion was used to induce a maximal state of coronary hyperemia at the discretion of the operator. FFR was calculated as the ratio of the calculated mean distal intracoronary pressure to the mean arterial pressure (15). Stenosis of ≥50% in the ICA was considered significant obstruction (16). A FFR value ≤0.80 was regarded as lesion-specific ischemia on a per-patient and per-vessel basis (16). FFR values between 0.75 and 0.8 were identified as the “gray zone” (17). We calculated the diagnostic accuracy of CT-FFR across the spectrum of conditions, such as ≤0.6, 0.60–0.69, 0.70–0.79, 0.80–0.89, and ≥0.90. All images and FFR signals were interpreted by two experienced interventional cardiologists who were blinded to the results of CTA and CT-FFR.

### 2.5. Study endpoints

The primary study endpoint was the assessment of sensitivity, specificity, area under the receiver-operating characteristic curve (AUC), diagnostic accuracy, positive predictive value (PPV), and negative predictive value (NPV) of CT-FFR on a per-patient and per-vessel basis, with invasive FFR (FFR ≤ 0.80) as the reference standard. Additionally, the diagnostic performance of coronary calcification was compared between subgroups stratified by the Agatston calcium score (AS), using a score of 400 as the grouping threshold. Patients were divided into a low-to-moderate calcification group (<400) and a high calcification group (≥400) (18). The diagnostic performance of “misdiagnosed” lesions was shown on a per-vessel basis.

### 2.6. Statistical analysis

The normality of quantitative data was assessed using the Kolmogorov–Smirnov test. Continuous variables are expressed as the mean ± standard deviation (SD) and were analyzed using the Student's *t*-test for normally distributed independent samples. Skewed data are expressed as the median (interquartile range, IQR) and were compared using non-parametric Mann–Whitney *U*-test. Categoric data are presented as frequencies and percentages and compared between groups with chi-squared or Fisher's test. The diagnostic performance characteristics of CT-FFR and CTA, with their corresponding 95% confidence intervals (CIs), were compared using the McNemar test and chi-square test. The AUC derived from receiver operating characteristic curve (ROC) analysis with invasive FFR (threshold: 0.80) as the reference standard was calculated, and comparisons were performed according to the method of DeLong et al. The Youden index was used to calculate the optimal threshold of CT-FFR for determining myocardial ischemia. Spearman's *r*-test correlation analysis and Bland–Altman analyses were used to examine the correlation between CT-FFR and FFR on a per-vessel level. Univariate logistic regression models were used to assess the effect of features on discordant lesions (false negative and false positive) in different CT-FFR categories. A two-tailed *P*-value <0.05 was considered significant. Statistical analyses were performed using R version 4.0.2.

## 3. Results

### 3.1. Patient characteristics

Of the 397 patients screened, six were excluded based on the inclusion and exclusion criteria (Figure 1). Therefore, a total of 324 vessels from 303 patients (median age: 62 years, IQR: 55–68 years; 63.7% men) had undergone CTA and ICA and were available for final analysis. Regarding CTA acquisition characteristics, the median heart rate was 72 bpm (IQR: 66–80). On CTA, the prevalence of CAD stenosis ≥50% on a per-vessel basis was 71%, with 153 (47.2) of these stenoses having an abnormal CT-FFR. The AS was calculated for 303 patients who were evaluated by CCTA, including 204 (67.3%) low to intermediate CACS (AS < 400) and 99 (32.7%) (AS ≥ 400) patients with high calcification. Invasive FFR was used to assess the presence of hemodynamically significant stenosis (FFR < 0.80) in 146 vessels (45.1%) of 141 patients (46.5%), with 238 (73.6%) lesions located in the LAD, 30 (9.2%) in the LCX, and 56 (17.2%) in the RCA. Additional baseline and lesion characteristics of CTA and FFR are presented in Table 1.

**Table 1:** Baseline characteristics and procedural results of per-patient and per-vessel.

Characteristic	*N*
Age, years	62 (55–68)
Male, *n* (%)	193 (63.7%)
Body mass index, kg/m^2^	24.9 (23.0–27.0)
Cardiovascular risk factors	
Diabetes, *n* (%)	89 (29.4%)
Hypertension, *n* (%)	174 (57.4%)
Dyslipidemia, *n* (%)	155 (51.2%)
Prior myocardial infarction, *n* (%)	4 (1.3%)
Stroke, *n* (%)	16 (5.3%)
Peripheral arteria disease, *n* (%)	15 (5.0%)
Tobacco abuse, *n* (%)	122 (40.3%)
Lesion characteristic	
Location (vessels)	324
LM/LAD, *n* (%)	238 (73.6%)
LCX, *n* (%)	30 (9.2%)
RCA, *n* (%)	56 (17.2%)
Invasive coronary angiography (ICA)	
Luminal stenosis degree ≥50%, *n* (%)	121 (37.3%)
Length of vessel lesion, mm	10.85 (6.92–15.15)
Lumen diameter of target lesion, mm	1.43 (1.13–1.70)
Resting Pd/Pa	0.93 (0.88–0.96)
FFR value (all patients)	0.82 (0.69–0.88)
FFR value (all vessels)	0.82 (0.70–0.88)
FFR value ≤ 0.80 on a per-patient basis, *n* (%)	141 (46.5%)
FFR value ≤ 0.80 on a per-vessel basis, *n* (%)	146 (45.1%)
CCTA parameters	
Heart rate, bpm	72 (66–80)
Luminal stenosis degree, *n* (%)	
30%–49%	94 (29.0%)
50%–69%	142 (43.8%)
70%–90%	88 (27.2%)
Area of vascular target lesion, mm^2^	1.87 (1.04–2.77)
Character of plaque, *n* (%)	
Fibrous plaque	148 (48.8%)
Calcified plaque	113 (37.3%)
Lipid plaque	124 (40.9%)
CT-FFR value (all patients)	0.81 (0.73–0.87)
CT-FFR value (all vessels)	0.81 (0.74–0.87)
CT-FFR ≤ 0.80 on a per-patient basis, *n* (%)	148 (48.8%)
CT-FFR ≤ 0.80 on a per-vessel basis, *n* (%)	153 (47.2%)
CAC score on patient basis (AS)	188.5 (21.3–563.3)
Low to intermediate CACS (AS < 400), *n* (%)	204 (67.3%)
High CACS (AS ≥ 400), *n* (%)	99 (32.7%)

CCTA, coronary computed tomography angiography; CT-FFR, fractional flow reserve derived from coronary computed tomography angiography; LM, left main artery; LAD, left anterior descending artery; LCX, Left circumflex artery; RCA, right coronary artery; FFR, fractional flow reserve; CAC score, coronary artery calcification score.

Categorical variables are expressed in absolute values and percentages; Quantitative variables were expressed as mean ± SD if normally distributed, Median and interquartile range (IQR) were provided for skewed data.

### 3.2. Diagnostic performance of CT-FFR on a per-patient basis and per-vessel basis

**Figure 3:**
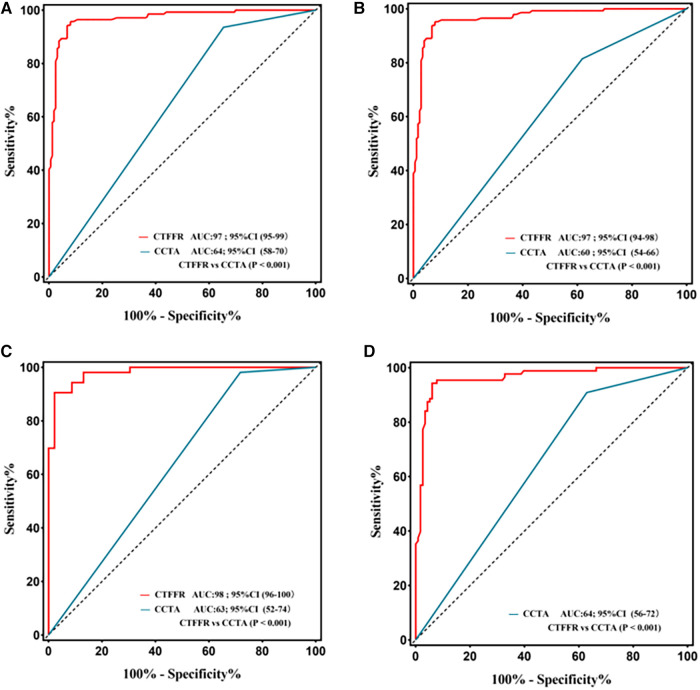
Receiver operating characteristic curve for diagnostic performance of CT-FFR and CCTA. The area under receiver operating characteristic curve (AUC) for the detection of ischemia by CT-FFR, and CCTA in lesions using invasive FFR as the reference standard; CCTA, coronary computed tomography angiography; CT-FFR, fractional flow reserve derived from coronary computed tomography angiography. (**A**) Per-patient; (**B**) Per-vessel; (**C)** Per patient in the high calcification group; (**D**) Per patient in the mild-to-moderate calcification.

The Youden index indicated an optimal threshold of 0.80 for CT-FFR to identify functionally ischemic lesions. The diagnostic sensitivity, specificity, accuracy, PPV, and NPV for CT-FFR on a per-patient basis were 96% (91%–98%), 92% (87%–96%), 94% (90%–96%), 91% (85%–95%), and 96% (92%–99%), respectively. On a per-vessel basis, the sensitivity, specificity, diagnostic accuracy, PPV, and NPV of CT-FFR for detecting functional stenosis (defined as invasive FFR < 0.80) were 95% (90%–98%), 92% (87%–96%), 94% (90%–96%), 91% (85%–95%), and 96% (92%–98%), respectively. The per-patient and per-vessel diagnostic accuracy, sensitivity, PPV, NPV, and for CT-FFR were higher than those for CCTA. The AUC of CT-FFR was higher than that for CCTA on per-patient basis and per-vessel basis (0.64 vs. 0.97, *P* < 0.001; 0.60 vs. 0.97, *P* < 0.001) (Figures 3A,B). The diagnostic performance of CCTA to detect hemodynamic stenosis in patients with suspected CAD is shown in Tables 2, 3.

**Table 2:** Diagnostic performance of CT-FFR, CCTA in all vessels.

All vessels (*n* = 324)	CCTA	CT-FFR	*P*-value
Sensitivity	82 (74–87)	95 (90–98)	<0.001
Specificity	62 (54–69)	92 (87–96)	<0.001
Accuracy	71 (65–76)	94 (90–96)	<0.001
PPV	64 (5–71)	91 (85–95)	<0.001
NPV	80 (73–87)	96 (92–98)	<0.001
AUC	60 (54–66)	97 (94–98)	<0.001

Values are% (95% confidence interval).

NPV, negative predictive value; PPV, positive predictive value; AUC, the area under receiver operating characteristic curve; CCTA, coronary computed tomography angiography; CT-FFR, fractional flow reserve derived from coronary computed tomography angiography.

**Table 3:** Diagnostic performance of CT-FFR, CCTA in all patients.

All patient (*n* = 303)	CCTA	CT-FFR	*P*-value
Sensitivity	94 (88–97)	96 (91–98)	0.595
Specificity	65 (58–73)	92 (87–96)	<0.001
Accuracy	79 (73–83)	94 (90–96)	<0.001
PPV	70 (63–77)	91 (85–95)	<0.001
NPV	92 (86–96)	96 (92–99)	0.257
AUC	64 (58–70)	97 (95–99)	<0.001

Values are% (95% confidence interval).

NPV, negative predictive value; PPV, positive predictive value; AUC, the area under receiver operating characteristic curve; CCTA, coronary computed tomography angiography; CT-FFR, fractional flow reserve derived from coronary computed tomography angiography.

### 3.3. Of coronary artery calcification on CT-FFR performance

The performance characteristics of CCTA and CT-FFR on a per-patient analysis across all AS categories are shown in Table 4 and Figures 3C,D. The ROC curves for CT-FFR showed superior diagnostic performance to CCTA in vessels with high AS (CAC ≥ 400, AUC: 0.98 vs. 0.63, *P* < 0.001) and low-to-intermediate AS (CAC >0 to <400, AUC: 0.96 vs. 0.64, *P* < 0.001). Compared to invasive FFR, CT-FFR was more accurate and specific than CCTA alone in patients with low to moderate or high AS.

**Table 4:** Diagnostic performance of CT-FFR and CCTA on a per-patient basis according to agatston score categories.

Patient (*n* = 324)	Low to intermediate CACS (*n* = 204)			High CACS (*n* = 99)		
+	CCTA	CT-FFR	*P*-value	CCTA	CT-FFR	*P*-value
Sensitivity	91 (83–96)	94 (87–98)	0.566	98 (90–100)	98 (90–100)	1.00
Specificity	37 (28–47)	94 (88–98)	<0.001	28 (16–43)	87 (74–95)	<0.001
Accuracy	57 (46–68)	93 (86–97)	<0.001	51 (35–65)	97 (87–99)	<0.001
PPV	52 (44–60)	92 (85–97)	<0.001	61 (50–72)	90 (79–96)	<0.001
NPV	84 (71–93)	96 (90–99)	0.024	93 (66–98)	98 (87–100)	0.44
AUC	64 (56–72)	96 (94–99)	<0.001	63 (52–74)	98 (96–100)	<0.001

Values are % (95% confidence interval).

NPV, negative predictive value; PPV, positive predictive value; AUC, the area under receiver operating characteristic curve; CCTA, coronary computed tomography angiography; CT-FFR, fractional flow reserve derived from coronary computed tomography angiography; CACS, coronary artery calcification scores.

### 3.4. Correlation of CT-FFR to FFR

**Figure 4:**
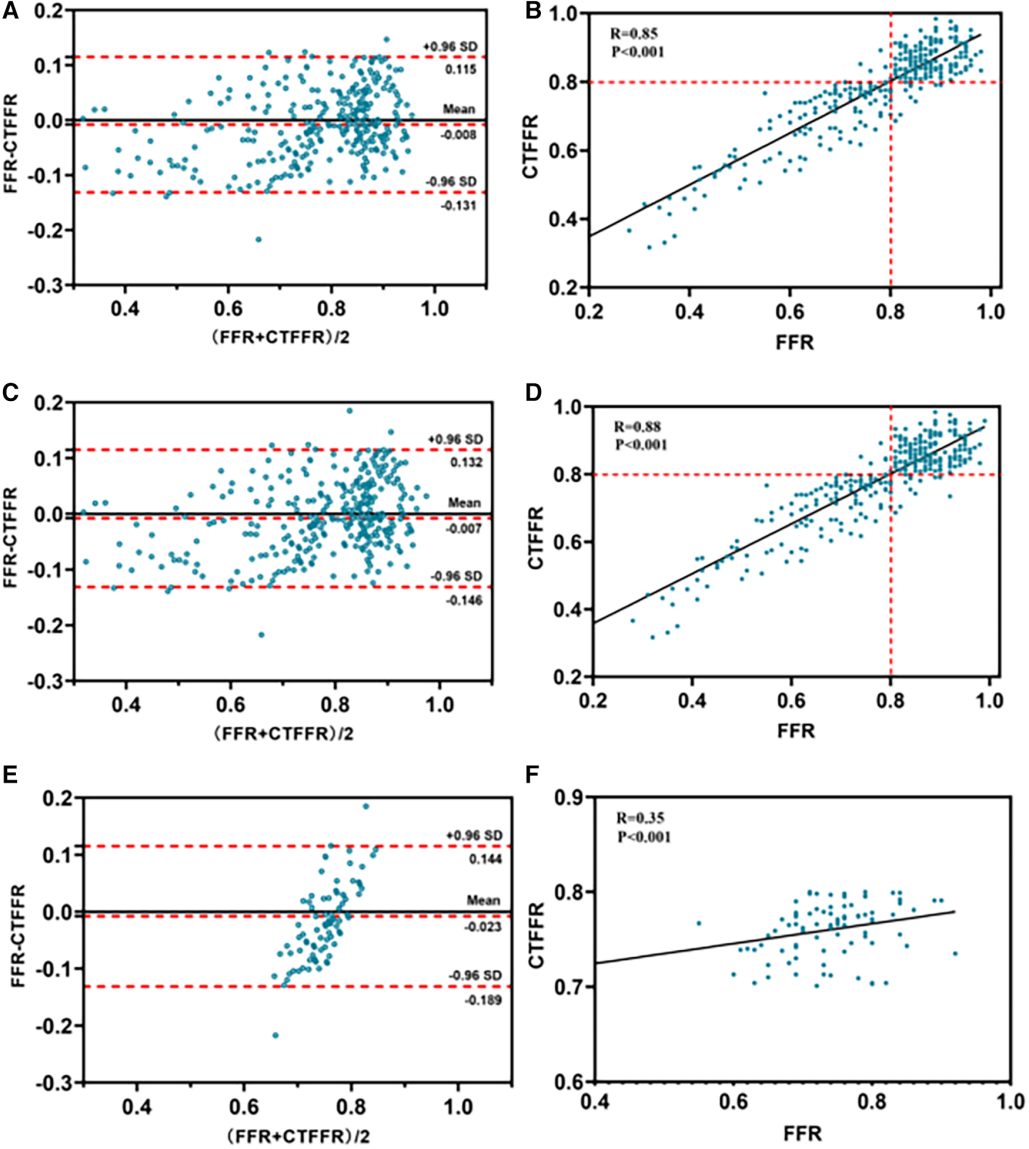
Bland-Altman and scatter plots for the association of CT-FFR and FFR. For all patients (*n* = 303) (panels **A**,**B**); for all vessels (*n* = 324) (panels **C**,**D**); for vessels with a CTFFR of 0.7–0.8 (*n* = 91) (panels **E**,**F**). CT-FFR, fractional flow reserve derived from coronary computed tomography angiography; FFR, fractional flow reserve.

The per-patient CT-FFR values showed a good correlation with FFR values (Spearman's rank correlation *r* = 0.85; *P* < 0.001), and per-vessel CT-FFR values showed a good correlation with FFR values (Spearman's rank correlation *r* = 0.88; *P* < 0.001) (Figure 4B,D). Bland–Altman analysis showed a slight underestimation of CT-FFR values compared to FFR on a per-patient basis (bias, −0.008; 95% limits of agreement: −0.131 to +0.115) and on a per-vessel basis (bias, −0.007; 95% limits of agreement: −0.146 to +0.132) (Figure 4A,C).

### 3.5. Agreement between CT-FFR and invasive FFR values

**Figure 5:**
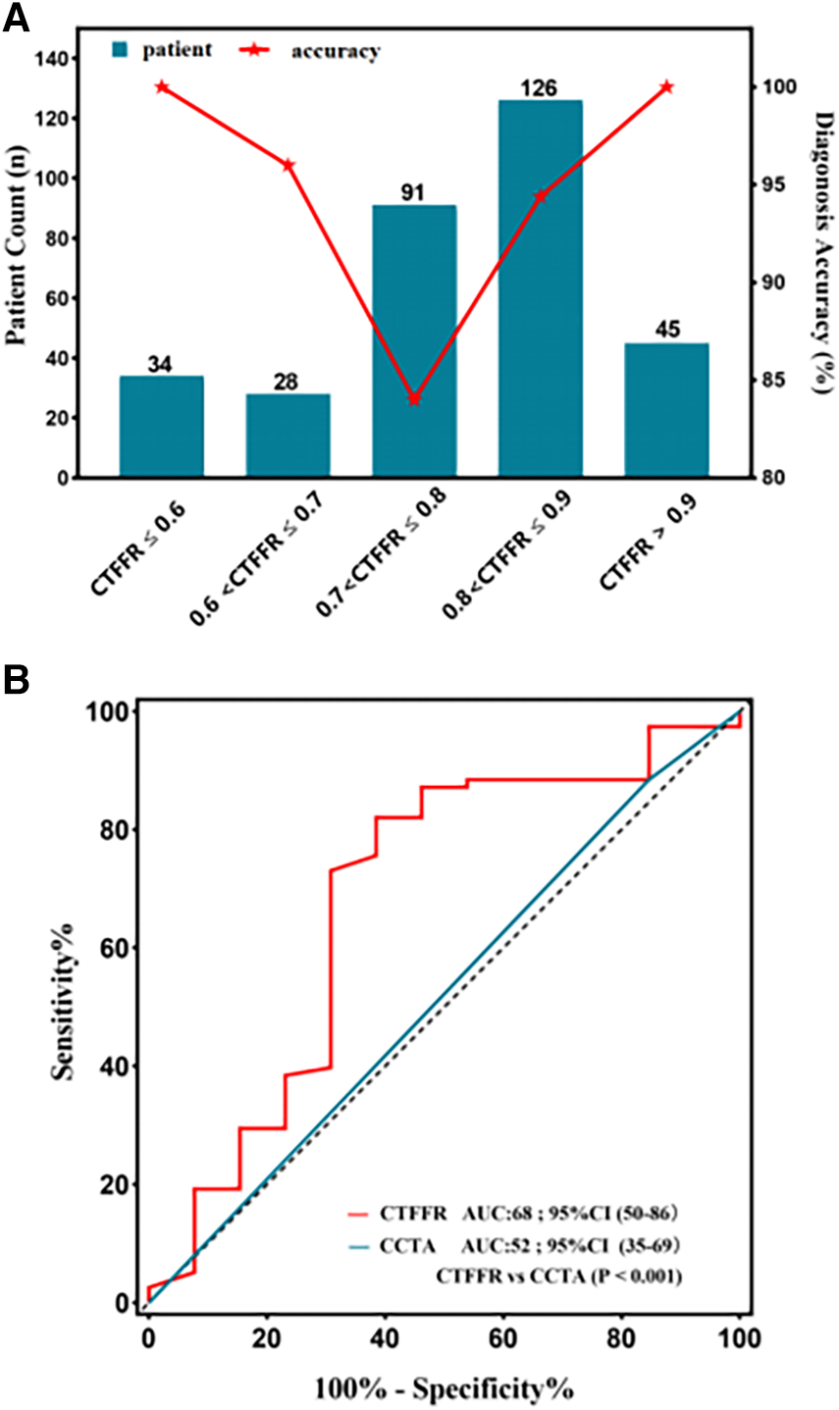
(**A**) diagnostic performance of CT-FFR in different categories. The diagnostic accuracy of lesions in CT-FFR (0.70–0.80) was the lowest, and the further from the zone, the higher the accuracy. (**B**) The area under the receiver operating characteristic curve (AUC) for detecting CT-FFR (0.7–0.8), the AUC was significantly lower in this interval compared to the overall.

Vessels with CT-FFR > 0.8 showed a significant increase in plaque burden parameters including target lesion lumen diameter, and vessel target lesion area size compared to vessels with CT-FFR (0.70–0.80) (all *P* < 0.05), while other plaque burden parameters showed no significant differences (Table 5). Very mild CT-FFR values (>0.90) provided almost complete certainty that the invasive FFR was negative for ischemia [44 of 45 (97.8%)]. Similarly, very severe CT-FFR values (≤0.60) provided a high degree of certainty that the invasive FFR was positive for ischemia [33 of 34 (97.0%)], albeit with fewer data points available for analysis at low CT-FFR values. However, nearer the cut point, there was less certainty, with classification agreement between invasive FFR and CT-FFR at its lowest in the CT-FFR 0.7–0.8 range (Figure 5A). The AUC for detecting CT-FFR (0.7–0.8) in this interval was significantly lower than the overall AUC (Figure 5B). Moreover, the linear correlation and scatter plots showed significant heteroskedasticity, with a significantly larger scatter for CT-FFR between 0.70 and 0.80 (*P* < 0.001) (Spearman's rank correlation *r* = 0.35; *P* < 0.001) (Figure 4F). Bland–Altman analysis demonstrated a small bias toward underestimation of invasive FFR by CT-FFR (bias, −0.023; *P *< 0.001), with 95% limits of agreement ranging from −0.189 to 0.144 (Figure 4E).

**Table 5:** Characteristics of coronary artery lesions at different CT-FFR levels.

	CT-FFR ≤ 0.70	*P*-value	0.70 < CT-FFR ≤ 0.8	*P*-value	CT-FFR > 0.08
Location (vessels)					
LAD, *n* (%)	43 (69.4%)	0.06	68 (83.9%)	0.01	117 (68.4%)
LCX, *n* (%)	2 (3.2%)	0.69	5 (6.2%)	0.13	23 (13.5%)
RCA, *n* (%)	17 (27.4%)	0.01	8 (9.9%)	0.09	31 (18.1%)
CCTA parameters					
Luminal stenosis degree ≥50%, *n* (%)	54 (87.0%)	<0.01	45 (49.5%)	<0.01	22 (12.9%),
Length of vessel lesion, mm	11.7 (± 5.7)	0.95	11.6 (± 6.1)	0.98	11.6 (± 6.6)
Lumen diameter of target lesion, mm	1.04 (± 0.41)	<0.01	1.36 (0.44)	<0.01	1.62 (± 0.44)
Area of vascular target lesion, mm^2^	1.10 (± 0.86)	<0.01	1.60 (± 1.05)	<0.01	2.60 (± 1.50)
Character of plaque, *n* (%)					
Fibrous plaque	34 (54.8%)	0.92	48 (52.7%)	0.11	71 (41.5%)
Calcified plaque	32 (51.6%)	0.30	38 (41.8%)	0.07	49 (28.7%)
Lipid plaque	23 (37.1%)	0.96	33 (36.3%)	0.08	82 (48.0%)
CAC score on patient	457.9 (474.1)	0.85	411.9 (509.9)	0.49	335.1 (515.3)

CCTA, coronary computed tomography angiography; CT-FFR, fractional flow reserve derived from coronary computed tomography angiography; CAC Score, coronary artery calcification score.

Categorical variables are expressed in absolute values and percentages; Quantitative variables were expressed as mean ± SD if normally distributed, Median and interquartile range (IQR) were provided for skewed data.

### 3.6. Univariate analysis for the prediction of mismatch findings on CT-FFR

Univariate logistic regression analysis was used to evaluate the effect of coronary characteristics on FN or FP lesions (Table 6). In all lesions, lumen stenosis degree ≥50% had a significant effect on decreasing the risk of FN and FP results of CT-FFR. In non-ischemia lesions (CTFFR *> *0.8), the lumen diameter (OR: 0.046, *P* = 0.03) of the target lesion had a significant negative effect on the risk of FP results of CT-FFR. Importantly, the diagnostic accuracy of CT-FFR was found to vary markedly across the spectrum of disease (Figure 5A).

**Table 6:** Effect of CCTA features on false-positive and false-negative lesions at different CT-FFR levels.

	CT-FFR ≤ 0.70		0.70 < CT-FFR ≤ 0.8		CT-FFR > 0.8	
	OR	*P*-value	OR	*P*-value	OR	*P*-value
LAD, *n* (%)	1.385	0.902	3.401	0.079	1.384	0.997
Lumen stenosis degree ≥50%, *n* (%)	0.160	<0.001	0.167	<0.001	0.043	<0.001
Length of vessel lesion, mm	1.085	0.582	0.946	0.328	0.984	0.792
Lumen diameter of target lesion, mm	3.752	0.559	1.127	0.865	0.046	0.016
Area of vascular target lesion, mm^2^	1.418	0.752	0.084	1.597	0.719	0.324
Calcified plaque	1.060	0.988	0.812	0.729	2.483	0.406
CAC score on patient	1.004	0.174	1.001	0.559	1.001	0.178

CCTA, coronary computed tomography angiography; CT-FFR, fractional flow reserve derived from coronary computed tomography angiography.

## 4. Discussion

This Chinese multicenter study demonstrated that CT-FFR based on novel CFD modeling has high diagnostic performance in the identification of ischemic lesions. We also provided evidence that coronary artery calcification did not significantly affect the diagnostic performance of CT-FFR. Even in the presence of severe calcification, CT-FFR was superior to CCTA alone. Given the significant variation in the diagnostic accuracy of CT-FFR across the spectrum of disease, our findings will allow clinicians to interpret the diagnostic accuracy of an individual FFR-CT result.

Coronary CTA can be effective in ruling out CAD in populations with low morbidity (19). However, the technique does not accurately interpret the hemodynamic severity of angiographic lesions. Consequently, computational solutions have been increasingly developed to assess the degree of flow obstruction on CTA. CFD and ML algorithms have been used in previous studies using noninvasive CT-FFR to detect functional coronary ischemia. For the CFD-based CT-FFR using the Siemens approach, the AUCs on a per-vessel basis ranged from 0.78–0.92 (18, 20, 21). For ML-based CT-FFR, the per-vessel AUCs ranged from 0.84–0.90 (22–24). Our study shows that the CT-FFR results are comparable, with an AUC of 0.97 on a per-patient and per-vessel basis, allowing the classification of functionally significant stenoses. These results demonstrate the feasibility of the CFD model to non-invasively determine the physiological consequences of CAD. The addition of CT-FFR to CCTA to measure anatomical stenosis could improve diagnostic accuracy and expand the utility of CCTA in this population.

A previous study demonstrated that the diagnostic accuracy of CCTA was higher in patients with calcification scores <600 compared to those with calcification scores >600, with diagnostic specificity increasing from 44% to 90%, and NPV increasing from 50% to 83% (25). However, recent studies have shown that CT-FFR combined with other hemodynamic indices, such as flow shear stress and flow velocity, may avoid overestimating calcified plaque by CCTA (26). Indeed, the NXT subgroup study showed that the differences in accuracy, sensitivity, and specificity of CT-FFR were not statistically significant between each interval group, and that CT-FFR had the same diagnostic efficacy (AUC: 0.86 and 0.92, respectively) (27). This was consistent with our findings that the sensitivity, specificity, and accuracy of CT-FFR between the low to intermediate calcification group and high calcification for detecting hemodynamically significant stenosis were 0.94 vs. 0.98, 0.94 vs. 0.87, and 0.93 vs. 0.97, respectively. Moreover, the AUC of CT-FFR was significantly higher than CCTA in the high calcification group (0.98 vs. 0.63) (*P* < 0.001). In patients with severe calcification, CT-FFR has a higher diagnostic value than CTA, with increasing specificity and PPV, while maintaining sensitivity and NPV, with high reproducibility. Other improvements in our CFD-based technique include using machine/deep learning for the geometric construction of vascular anatomy, adaptive coronary and aortic meshing, and simulation under steady-state flow conditions. The average time to obtain CT-FFR calculations using our technique is 10 min, which is acceptable in a clinical setting.

Lesions located in the “gray area” have been challenging for CT-FFR, and diagnostic performance in this area will inevitably decrease; however, current guidelines do not clearly define the gray area (9, 28). In one study, when the FFR-CT value was <0.63 or >0.83, the diagnostic accuracy threshold was 82% (overall), while more stringent diagnostic accuracy thresholds of 95% and 98% were met with FFR-CT values <0.53 or >0.93 and <0.47 or >0.99, respectively (13). In this current study, we found that the diagnostic accuracy decreased as expected as the reference measure (CT-FFR) approached the diagnostic threshold. In the presence of ischemia, very mild FFR-CT values (>0.90) almost completely identified negative invasive FFR. Similarly, very severe CT-FFR values (<0.60) identified invasive FFR ischemia as positive, despite fewer data points being available for analysis at low FFR-CT values. However, the certainty was lower nearer the cut point, with the lowest concordance between invasive FFR and CT-FFR for classification between 0.7 and 0.8 (AUC = 0.68). In this study, the prevalence of physiologically intermediate stenoses (CT-FFR, 0.70–0.80) was 28.1% (91/324). Our study provided an illustration of the diagnostic accuracy of an individual CT-FFR result that may be available in clinical practice when a higher level of accuracy is required. Physicians and patients can balance the degree of uncertainty with the need for invasive confirmation of ischemia and the need to determine the range of FFR values that meet an acceptable level of diagnostic accuracy.

The CREDENCE trial reported a strong correlation between volumetric measurements of atherosclerotic plaques, especially non-calcified plaques, and lumen size, which was significantly associated with FFR (29). Recent studies have reported significantly lower FFR results with CT findings consistent with plaque vulnerability (such as positive remodeling and low attenuation), and even with similar stenosis (30, 31). The use of an integrated plaque assessment of CCTA, rather than just luminal stenosis, contributes to an important difference between our results and other research. Compared to stenosis assessment alone, plaque assessment and FFR-CT provide better discrimination of ischemia (32). Our further analysis of misdiagnosed CT-FFR lesions showed that for physiologically intermediate stenosis lesions (CT-FFR, 0.70–0.80), calcified plaque had no significant effect on increasing the risk of FN CT-FFR. Moreover, the diameter of the target lesion had a significant effect on the reduction in the risk of FP results in non-ischemia lesions (CT-FFR *> *0.8). Furthermore, when the degree of stenosis is >50%, the incidence of FP and FN results at different intervals of the CT-FFR is reduced. CT-FFR did not significantly improve the discrimination between normal and abnormal FFR, supporting the concept of reduced pressure difference as a response to specific underlying atherosclerotic plaque characteristics. The results of the consistency analysis demonstrated that affecting CT-FFR in the interval 0.70–0.80 were more problematic than the disagreement with FFR, and most of them were concentrated in lesions with moderate stenosis. Additionally, the influence of vascular characteristics on the diagnostic power of CT-FFR was unrelated to whether it was associated with calcified plaque, which differed significantly from CTA. These findings highlight the complex, multifaceted nature of coronary ischemia, which is influenced by the characteristics of the coronary lumen analyzed during CT-FFR derivation.

## 5. Limitations

This study has several limitations that warrant discussion. First, although the small sample size of patients with severe coronary calcification is consistent with the real world, a larger study population with close similarity to the real clinical situation, especially patients with CAC ≥ 400 or even ≥1,000, must confirm the results. Although the diagnostic performance of CT-FFR on calcified vessels was much better than the older CT-FFR algorithm, the degree of coronary calcium had a small but significant impact on its accuracy.

Second, the studies we reviewed excluded some patients after they underwent a CT scan because these images were considered inappropriate for calculating the FFR. The technical and clinical capabilities of FFR-CT will likely continue to improve in the future.

Finally, net reclassification analysis is another way to characterize the clinical utility of CT-FFR. However, in the absence of patient-specific outcomes for FFR-CT values, no such analysis is currently possible. Individualized outcomes (in terms of both invasive FFR and CT-FFR values) for net reclassification analysis may be useful in future studies on the diagnostic performance of CT-FFR.

## 6. Conclusions

The results of this prospective, multicenter clinical study validated the diagnostic performance of the software for assessing coronary physiological function in coronary artery stenosis lesions, which was superior to CCTA, independent of calcified plaque. The diagnostic accuracy of CT-FFR varies widely across the disease spectrum, and we determined the impact of CT-FFR in the gray area (0.70–0.80), which is a diagnostic demarcation line. This information can be applied by clinicians using CT-FFR, in conjunction with patient-specific factors, to determine when the costs and risks of invasive angiography can be safely avoided. Future research should support the broader inclusion of atherosclerotic plaque findings that have implications for assessing coronary physiology, including the incorporation of plausible findings in clinical practice.

## Data Availability

The raw data supporting the conclusions of this article will be made available by the authors, without undue reservation.

## References

[1] Leipsic J Abbara S Achenbach S Cury R Earls JP Mancini GBJ SCCT guidelines for the interpretation and reporting of coronary CT angiography: a report of the society of cardiovascular computed tomography guidelines committee. J Cardiovasc Comput Tomogr. (2014) 8(5):342–58. 10.1016/j.jcct.2014.07.003 25301040

[2] Budoff MJ Dowe D Jollis JG Gitter M Sutherland J Halamert E Diagnostic performance of 64-multidetector row coronary computed tomographic angiography for evaluation of coronary artery stenosis in individuals without known coronary artery disease: results from the prospective multicenter ACCURACY (assessment by coronary computed tomographic angiography of individuals undergoing invasive coronary angiography) trial. J Am Coll Cardiol. (2008) 52(21):1724–32. 10.1016/j.jacc.2008.07.031 19007693

[3] Driessen RS Danad I Stuijfzand WJ Raijmakers PG Schumacher SP van Diemen PA Comparison of coronary computed tomography angiography, fractional flow reserve, and perfusion imaging for ischemia diagnosis. J Am Coll Cardiol. (2019) 73(2):161–73. 10.1016/j.jacc.2018.10.056 30654888

[4] Dey D Lee CJ Ohba M Gutstein A Slomka PJ Cheng V Image quality and artifacts in coronary CT angiography with dual-source CT: initial clinical experience. J Cardiovasc Comput Tomogr. (2008) 2(2):105–14. 10.1016/j.jcct.2007.12.017 19083930

[5] Montalescot G Sechtem U Achenbach S Andreotti F Arden C Budaj A 2013 ESC guidelines on the management of stable coronary artery disease: the task force on the management of stable coronary artery disease of the European Society of Cardiology. Eur Heart J (2013) 34(38):2949–3003. 10.1093/eurheartj/eht296 23996286

[6] Zimmermann FM Ferrara A Johnson NP van Nunen LX Escaned J Albertsson P Deferral vs. Performance of percutaneous coronary intervention of functionally non-significant coronary stenosis: 15-year follow-up of the DEFER trial. Eur Heart J. (2015) 36(45):3182–8. 10.1093/eurheartj/ehv452 26400825

[7] Barbato E Toth GG Johnson NP Pijls NHJ Fearon WF Tonino PAL A prospective natural history study of coronary atherosclerosis using fractional flow reserve. J Am Coll Cardiol. (2016) 68(21):2247–55. 10.1016/j.jacc.2016.08.055 27884241

[8] Chinnaiyan KM Safian RD Gallagher ML George J Dixon SR Bilolikar AN Clinical use of CT-derived fractional flow reserve in the emergency department. JACC Cardiovasc Imaging. (2020) 13(2):452–61. 10.1016/j.jcmg.2019.05.025 31326487

[9] Nakazato R Park H-B Berman DS Gransar H Koo B-K Erglis A Noninvasive fractional flow reserve derived from computed tomography angiography for coronary lesions of intermediate stenosis severity: results from the DeFACTO study. Circ Cardiovasc Imaging. (2013) 6(6):881–9. 10.1161/circimaging.113.000297 24081777

[10] Koo BK Erglis A Doh JH Daniels DV Jegere S Kim HS Diagnosis of ischemia-causing coronary stenoses by noninvasive fractional flow reserve computed from coronary computed tomographic angiograms. Results from the prospective multicenter DISCOVER-FLOW (diagnosis of ischemia-causing stenoses obtained via noninvasive fractional flow reserve) study. J Am Coll Cardiol. (2011) 58(19):1989–97. 10.1016/j.jacc.2011.06.066 22032711

[11] Norgaard BL Leipsic J Gaur S Seneviratne S Ko BS Ito H Diagnostic performance of noninvasive fractional flow reserve derived from coronary computed tomography angiography in suspected coronary artery disease: the NXT trial (analysis of coronary blood flow using CT angiography: next steps). J Am Coll Cardiol. (2014) 63(12):1145–55. 10.1016/j.jacc.2013.11.043 24486266

[12] Lu MT Ferencik M Roberts RS Lee KL Ivanov A Adami E Noninvasive FFR derived from coronary CT angiography. JACC Cardiovasc Imaging. (2017) 10(11):1350–8. 10.1016/j.jcmg.2016.11.024 28412436 PMC5632098

[13] Cook CM Petraco R Shun-Shin MJ Ahmad Y Nijjer S Al-Lamee R Diagnostic accuracy of computed tomography–derived fractional flow reserve: a systematic review. JAMA Cardiol. (2017) 2(7):803. 10.1001/jamacardio.2017.1314 28538960

[14] Mittal R Dong H Bozkurttas M Najjar FM Vargas A von Loebbecke A. A versatile sharp interface immersed boundary method for incompressible flows with complex boundaries. J Comput Phys. (2008) 227(10):4825–52. 10.1016/j.jcp.2008.01.028 20216919 PMC2834215

[15] Toth GG Johnson NP Jeremias A Pellicano M Vranckx P Fearon WF Standardization of fractional flow reserve measurements. J Am Coll Cardiol. (2016) 68(7):742–53. 10.1016/j.jacc.2016.05.067 27515335

[16] Tonino PAL De Bruyne B Pijls NHJ Siebert U Ikeno F van `t Veer M Fractional flow reserve versus angiography for guiding percutaneous coronary intervention. N Engl J Med (2009) 360(3):213–24. 10.1056/nejmoa0807611 19144937

[17] Xaplanteris P Fournier S Pijls NHJ Fearon WF Barbato E Tonino PAL Five-year outcomes with PCI guided by fractional flow reserve. N Engl J Med. (2018) 379(3):250–9. 10.1056/nejmoa1803538 29785878

[18] Agatston AS Janowitz WR Hildner FJ Zusmer NR Viamonte M Jr Detrano R. Quantification of coronary artery calcium using ultrafast computed tomography. J Am Coll Cardiol. (1990) 15(4):827–32 (新添加). 10.1016/0735-1097(90)90282-t 2407762

[19] Newby DE Adamson PD Berry C Boon NA Dweck MR Williams MC. Coronary CT angiography and 5-year risk of myocardial infarction. N Engl J Med. (2018) 379(10):924–33. 10.1056/NEJMoa1805971 30145934

[20] Nørgaard BL Hjort J Gaur S Hansson N Bøtker HE Leipsic J Clinical use of coronary CTA–derived FFR for decision-making in stable CAD. JACC Cardiovasc Imaging. (2017) 10(5):541–50. 10.1016/j.jcmg.2015.11.025 27085447

[21] Coenen A Lubbers MM Kurata A Kono A Dedic A Chelu RG Fractional flow reserve computed from noninvasive CT angiography data: diagnostic performance of an on-site clinician-operated computational fluid dynamics algorithm. Radiology. (2015) 274(3):674–83. 10.1148/radiol.14140992 25322342

[22] Itu L Rapaka S Passerini T Georgescu B Schwemmer C Schoebinger M A machine-learning approach for computation of fractional flow reserve from coronary computed tomography. J Appl Physiol. (2016) 121(1):42–52. 10.1152/japplphysiol.00752.2015 27079692

[23] Tesche C De Cecco CN Baumann S Renker M McLaurin TW Duguay TM Coronary CT angiography–derived fractional flow reserve: machine learning algorithm versus computational fluid dynamics modeling. Radiology. (2018) 288(1):64–72. 10.1148/radiol.2018171291 29634438

[24] Coenen A Kim Y-H Kruk M Tesche C De Geer J Kurata A Diagnostic accuracy of a machine-learning approach to coronary computed tomographic angiography–based fractional flow reserve: result from the MACHINE consortium. Circ Cardiovasc Imaging. (2018) 11(6):e007217. 10.1161/circimaging.117.007217 29914866

[25] Feldman DI Latina J Lovell J Blumenthal RS Arbab-Zadeh A. Coronary computed tomography angiography in patients with stable coronary artery disease. Trends Cardiovasc Med. (2022) 32(7):421–8. 10.1016/j.tcm.2021.08.009 34454051

[26] Fu D Xiao X Gao T Feng L Wang C Yang P Effect of calcification based on computer-aided system on CT-fractional flow reserve in diagnosis of coronary artery lesion. Comput Math Methods Med. (2022) 2022:1–10. 10.1155/2022/7020209 PMC8786524 35082914

[27] Nørgaard BL Gaur S Leipsic J Ito H Miyoshi T Park S-J Influence of coronary calcification on the diagnostic performance of CT angiography derived FFR in coronary artery disease. JACC Cardiovasc Imaging. (2015) 8(9):1045–55. 10.1016/j.jcmg.2015.06.003 26298072

[28] Schuijf JD Ko BS Di Carli MF Hislop-Jambrich J Ihdayhid A-R Seneviratne SK Fractional flow reserve and myocardial perfusion by computed tomography: a guide to clinical application. Eur Heart J Cardiovasc Imaging. (2018) 19(2):127–35. 10.1093/ehjci/jex240 29045612

[29] Stuijfzand WJ van Rosendael AR Lin FY Chang H-J van den Hoogen IJ Gianni U Stress myocardial perfusion imaging vs coronary computed tomographic angiography for diagnosis of invasive vessel-specific coronary physiology. JAMA Cardiol. (2020) 5(12):1338–48. 10.1001/jamacardio.2020.3409 32822476 PMC7439215

[30] Park H-B Heo R ó Hartaigh B Cho I Gransar H Nakazato R Atherosclerotic plaque characteristics by CT angiography identify coronary lesions that cause ischemia: a direct comparison to fractional flow reserve. JACC Cardiovasc Imaging. (2015) 8(1):1–10. 10.1016/j.jcmg.2014.11.002 25592691 PMC4297319

[31] Ahmadi A Leipsic J Øvrehus KA Gaur S Bagiella E Ko B Lesion-specific and vessel-related determinants of fractional flow reserve beyond coronary artery stenosis. JACC Cardiovasc Imaging. (2018) 11(4):521–30. 10.1016/j.jcmg.2017.11.020 29311033

[32] Gaur S Øvrehus KA Dey D Leipsic J Bøtker HE Jensen JM Coronary plaque quantification and fractional flow reserve by coronary computed tomography angiography identify ischaemia-causing lesions. Eur Heart J. (2016) 37(15):1220–7. 10.1093/eurheartj/ehv690 26763790 PMC4830909

